# Effects of Dual-Task Stroboscopic Visual Training on Balance, Functional Mobility, and Gait in Children Who Are Hard-of-Hearing: A Exploratory Randomized Controlled Study

**DOI:** 10.3390/jcm14248736

**Published:** 2025-12-10

**Authors:** Hafiza Gözen, Serkan Usgu, Yavuz Yakut

**Affiliations:** 1 Vocational School of Health Services, Gaziantep University, 27310 Gaziantep, Türkiye; 2 Faculty of Health Sciences, Hasan Kalyoncu University, 27010 Gaziantep, Türkiye; serkan.usgu@hku.edu.tr (S.U.); yavuz.yakut@hku.edu.tr (Y.Y.)

**Keywords:** hard-of-hearing children, balance, postural sway

## Abstract

**Objective**: This study aimed to investigate the effects of dual-task stroboscopic visual training (DTSVT) on balance, functional mobility, and gait in children who are hard-of-hearing. **Methods**: This randomized controlled study included 31 children (17 girls, 14 boys) with congenital sensorineural hearing loss. Participants were assigned to one of three groups: control group, conventional balance training (CBT) group, and DTSVT group. The CBT and DTSVT groups participated in an exercise program for 16 weeks, twice weekly, for 40 min (a total of 24 sessions). Static balance was assessed using the Tandem Romberg test and Single-Leg Stance (SLS) test, while dynamic balance was evaluated using the Functional Reach Test (FRT), balance disc test, and the Four Square Step Test (FSST). The Pediatric Balance Scale (PBS) was used as a subjective balance assessment. Functional mobility was assessed using the Timed Up and Go (TUG) Test, Step Test, 10 m Walk Test (10 MWT), and Functional Gait Assessment (FGA). Postural sway parameters were recorded using the GyKo device, including Sway Area (EA, cm^2^), Distance Length (DL, cm), Length (anterior–posterior (AP)) (cm), Length (medial–lateral (ML)) (cm), Mean Distance (D) (cm), Mean Distance (AP) (cm), and Mean Distance (ML) (cm). **Results**: Significant between-group differences were primarily observed in favor of the DTSVT group post-treatment, particularly in PBS scores, GyKoDL values during the eyes-open SLS test, and TUG test completion times (*p* < 0.05). Some baseline differences were noted among groups in functional reach distance, FSST completion time, and eyes-closed duration on the Balance Disc test (*p* < 0.05). Within-group comparisons revealed significant improvements in FSST times in both intervention groups, reduced postural sway parameters during the FRT in the DTSVT and control groups, and increased eyes-closed Tandem Romberg duration in the CBT group (*p* < 0.05). Most other outcome measures did not demonstrate statistically significant changes either within or between groups (*p* > 0.05). **Conclusions**: Dual-task stroboscopic visual training was more effective than conventional balance training in improving specific aspects of balance and functional mobility in children who are hard-of-hearing. These findings highlight the potential of adding cognitively demanding and visually engaging balance tasks to rehabilitation programs for this population. Larger and more diverse samples in future studies are needed to enhance the generalizability of these results. Studies that assess balance and gait using standardized clinical or laboratory tests may be particularly valuable. Given the small sample size and multiple comparisons, the results should be considered preliminary and exploratory.

## 1. Introduction

Hearing loss in childhood can affect not only communication and language development but also motor performance and balance. Previous research has shown that children who are hard-of-hearing may face challenges in general dynamic coordination, visuomotor coordination, and movement speed [1,2]. These motor outcomes cannot be attributed solely to hearing loss, but also emerge within the broader cognitive ecology in which these children are raised, including environmental, social, and educational factors [3]. Furthermore, studies have shown that children with hearing loss experience vestibular disorders and a reduction in balance-related quality of life [4,5]. Vestibular dysfunction in children can lead to symptoms such as imbalance, gait disturbances, falls, collisions, and dizziness. These symptoms can hinder engagement in age-appropriate neuromotor activities, such as playing games or using playground equipment [6,7]. Early diagnosis and intervention can reduce the disparities in motor skills observed between children who are hard-of-hearing and their typically developing peers [1]. Children who are hard-of-hearing are not routinely assessed by clinicians for balance or motor development difficulties unless they have a diagnosed neurological or orthopedic condition. However, parents and teachers frequently report concerns about incoordination and balance problems that adversely affect these children’s daily activities and school performance [8].

In a previous study, children who are hard-of-hearing and their peers with normal hearing underwent a 12-session exercise program that incorporated both static and dynamic balance exercises, as well as activities aimed at enhancing somatosensory awareness. Post-intervention assessments revealed improvements in balance among the children who are hard-of-hearing, leading to the conclusion that exercise programs targeting somatosensory function may effectively enhance postural control in this population [9]. In another study, children with sensorineural hearing impairment were assigned to one of three groups: control, Tai Chi, or conventional exercise. Following a 10-week training program conducted twice weekly, both the Tai Chi and conventional exercise groups demonstrated significant improvements in balance performance [10]. While educational and rehabilitation programs for individuals who are hard-of-hearing have traditionally emphasized somatosensory-based exercises and games, there remains a notable gap in interventions targeting dual-task paradigms or the integration of visual system training for balance enhancement.

The stroboscopic effect is a visual phenomenon that occurs when continuous rotational or other cyclic motion is represented by a series of brief or instantaneous samples. This phenomenon has been incorporated into training programs for sports activities popular among young adults, including basketball and football [11]. By forcing reliance on limited visual information, stroboscopic training enhances perceptual and attentional faculties that underpin fundamental visuomotor control. Research on healthy adults has demonstrated that stroboscopic training improves temporal anticipation, visual cognition, visual attention, and perceptual abilities [12]. This training employs specialized glasses equipped with transparent lenses that permit full vision and opaque lenses that intermittently obstruct vision by automatically blinking, thereby producing the intended stroboscopic effect [13].

The maintenance and regulation of postural balance require substantial information-processing resources; consequently, more complex motor tasks may impose demands that exceed the available capacity [14]. Dual-task exercises, grounded in neurophysiological principles, involve the simultaneous execution of cognitive and motor tasks [15,16]. Dual-task paradigms emphasize two critical dimensions: the attentional resources required for motor task execution and the impact of concurrently performing cognitive and motor tasks on motor performance [17].

Although somatosensory-based training has been commonly used in interventions for children who are hard-of-hearing, there is limited evidence on the combined effect of dual-task and stroboscopic visual training on postural control in this population. This study focused on children who are hard-of-hearing, a population in which the presence of balance disorders remains a matter of debate and for which high-quality research is limited. This uncertainty is also reflected in clinical practice, where routine interventions or training targeting balance deficits are not provided unless these concerns are explicitly reported by the child or their family. The aim of this study was to examine the impact of integrating dual-task training and stroboscopic visual training alongside conventional balance exercises on balance, functional mobility, and overall gait parameters in children who are hard-of-hearing. We hypothesized that combining dual-task stroboscopic visual training with conventional balance exercises would yield superior improvements in balance and functional mobility compared to conventional balance training alone.

## 2. Methods

### 2.1. Study Design

This randomized controlled, three-arm parallel study adhered to the CONSORT guidelines for non-pharmacological trials. Children diagnosed with congenital sensorineural hearing loss who met the inclusion criteria were allocated by simple randomization into one of three groups: control, conventional balance training (CBT), and dual-task stroboscopic visual training (DTSVT). Randomization was performed using a computer-generated simple random number list by an independent researcher not involved in assessments or interventions. Allocation was concealed with sequentially numbered, opaque sealed envelopes. Assessments were conducted at baseline and at 16 weeks post-intervention. Both the CBT and DTSVT groups participated in a structured exercise program comprising 24 sessions, conducted twice weekly, with each session lasting 40 min. The control group received no intervention and continued with their usual activities throughout the study period.

The study was conducted in centers providing auditory rehabilitation and services for speech and language disorders located in Gaziantep, Turkey. Following the receipt of the necessary institutional approvals and the procurement of required equipment, the assessment phase commenced on 28 December 2021. Evaluation, intervention, and follow-up procedures continued through 4 April 2023.

### 2.2. Participants

**Figure 1:**
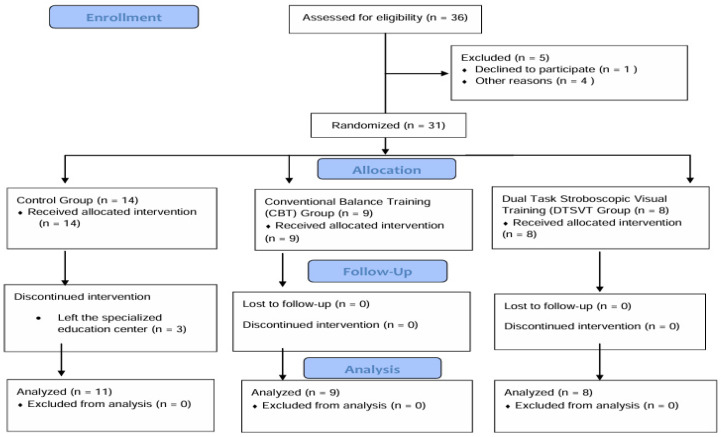
Study flow diagram.

The study enrolled 31 children (17 girls, 14 boys), aged 7–12 years, who were diagnosed with congenital bilateral sensorineural hearing loss by an otolaryngologist. Children were included in the study if they had not undergone cochlear implant surgery, were able to walk independently, and had a hearing threshold of 71–90 dB based on audiological tests. Children with additional health conditions were excluded, including those with balance disorders of neurological origin. Moreover, children with cochlear implants were not recruited to minimize the risk of vestibular dysfunction associated with surgical complications. Participants meeting any of the following criteria were excluded from the study: (i) any physical or visual impairment, (ii) lack of cooperation for the intervention, and (iii) neurological or systemic conditions potentially affecting balance [18,19]. The children participating in the study were able to hear sounds clearly while wearing hearing aids. They also had no difficulty speaking, except for some pronunciation of words. The study flow diagram is presented in Figure 1.

The study was conducted in accordance with the ethical principles outlined in the Declaration of Helsinki. Prior to initiation of the study, all participants and their parents were thoroughly informed about the study’s purpose and procedures involved and their approvals were obtained. Ethical approval was granted by the Non-Invasive Research Ethics Committee of Hasan Kalyoncu University Faculty of Health Sciences (No. 2020/115, Date: 16 December 2020). This study was supported by the Scientific and Technological Research Council of Turkey (TÜBİTAK) under project number 222S192. The study is registered on ClinicalTrials.gov (ID: NCT05404126).

### 2.3. Assessments

A single physiotherapist conducted both the intervention and outcome assessments. To avoid bias, participants were unaware of which treatment they received, and the statistical analysis was conducted by another researcher who was unaware of the participants. At baseline, participants’ physical characteristics, including hearing threshold (dB) values, age (years), height (cm), and body weight (kg), were recorded. The most appropriate static and dynamic balance, functional mobility and walking tests for children reported in the literature were selected. Static balance was assessed using the Tandem Romberg test and Single-Leg Stance (SLS) test, while dynamic balance was evaluated using the Functional Reach Test (FRT), balance disc test, and the Four Square Step Test (FSST). The Pediatric Balance Scale (PBS) was used as a subjective balance assessment. Functional mobility was assessed using the Timed Up and Go (TUG) Test, Step Test, 10 m Walk Test (10 MWT), and Functional Gait Assessment (FGA). The GyKo device, which enables quantitative evaluation of postural sway, was used to provide a more objective assessment of these tests. Furthermore, tests were performed three times for each participant, and the continuation time of the tests was recorded. To reduce the influence of extreme values, the median value of the three trials was considered for further analysis [20].

#### 2.3.1. Balance

Balance was assessed using both static and dynamic measures.

Static balance was evaluated using the Tandem Romberg test and the SLS test. For the Tandem Romberg test, participants were instructed to stand upright with one foot placed directly in front of the other, with arms crossed over their shoulders. For the SLS test, participants stood on their preferred leg with hands placed on their hips. The duration of maintaining balance was recorded for each test in seconds, with the average of three trials used for analysis.

Dynamic balance was assessed using the FRT, standing balance test on a balance disc, and the FSST. For the FRT, the child was asked to stand sideways next to a measuring tape affixed to the wall at shoulder height, with their right arm positioned close to but not touching the wall. The initial distance between the acromion and the third metacarpal was recorded. Then, the child was asked to reach forward horizontally with the arm extended as far as possible. The difference between the initial and final reach positions was measured in centimeters. For the standing balance test, the child was asked to stand on a compliant surface (balance disc) with eyes open and then eyes closed, and sway in the anterior–posterior and medial–lateral directions was observed. The duration of standing in each position was recorded in seconds. The FSST was used to assess the child’s ability to step forward, backward, and sideways. The time taken to complete the sequence was recorded in seconds. Additionally, balance was subjectively assessed using the PBS, scored item by item [21,22,23,24,25].

#### 2.3.2. Functional Mobility

Functional mobility was assessed using the TUG test and the Step Test, with results recorded in seconds. For the TUG test, the child was instructed to stand up from a chair, walk 3 m, turn around, walk back, and sit down [26]. Functional mobility was also assessed using a timed single-step test, where participants were instructed to ascend and descend a single step as quickly and safely as possible. The total time taken to complete the task was recorded using a stopwatch [27,28].

#### 2.3.3. Gait

Gait was assessed using the 10 MWT and the FGA. For the 10 MWT, the child was asked to walk a 10 m distance on a flat surface at a normal pace. Timing was recorded between the 2nd and 8th meters using a stopwatch, and the duration was recorded in seconds [29]. The FGA is used to evaluate functional walking in children. It includes 10 tasks, such as normal walking, fast walking, walking with head turns, and over obstacles. Each task is scored 0–3. The test is conducted under clinical supervision and takes approximately 10–15 min [30].

### 2.4. Postural Sway

In addition to recording time- or distance-based outcomes from the balance, gait, and functional mobility tests (e.g., FRT), postural sway during these tests was measured using the GyKo system (Microgate, Bolzano, Italy). Gyko is a measurement device specifically designed for motion analysis that uses validated Microgate software (version: 1.1.1.6) algorithms to describe the kinematics of the analyzed body segment and provide immediate feedback on the execution and quality of the physical movement [31]. The system is equipped with a range of supports to ensure optimal positioning and secure attachment of the device. Data are transferred to a computer via Bluetooth 4.0 connection for data processing and interpretation [32]. The GyKo system demonstrated good to excellent intra-rater reliability (ICCs = 0.84–0.95) and inter-rater reliability (ICCs = 0.82–0.94), as well as excellent concurrent validity compared to the optical motion system (ICCs = 0.90–0.95) [33].

For GyKo measurements, the system was attached to the participant’s back at the T1–T2 vertebral level by palpating the spinous processes. A special elastic strap was used, with the tension of the fastening bands individually adjusted for each participant. The vertical height of the sensor from the ground was determined based on the specific test performed and entered into the system. Before each test, the participant was asked to remain as still as possible. During postural sway assessments, the following parameters recorded by the device were used: total sway area (EA, cm^2^), defined as the ellipse encompassing approximately 95% of the trajectory points; total distance traveled (Distance Length (DL), cm); total distance traveled in the anterior–posterior direction (Length (AP), cm); total distance traveled in the medial–lateral direction (Length (ML), cm); mean distance from the center of the trajectory (Mean Distance (D), cm); mean distance from the center in the anterior–posterior direction (Mean Distance (AP), cm); and mean distance from the center in the medial–lateral direction (Mean Distance (ML), cm). Data were collected and transmitted wirelessly to a computer in real time. Statistical analyses were conducted on the collected data. To minimize table overload, only key outcomes were tabulated; significant differences in certain postural sway parameters, such as the DL value, were reported in the text rather than included in tables.

### 2.5. Exercise Training

The conventional balance training (CBT) and dual-task stroboscopic visual training (DTSVT) groups participated in a 16-week structured exercise program consisting of 24 sessions, conducted twice weekly, each lasting 40 min. The control group did not receive any exercise training. The specific exercises provided to the CBT group are detailed in Table 1 [10].

In the DTSVT group, children performed motor–motor and motor–cognitive dual-task exercises in addition to conventional balance exercises. Visionup stroboscopic glasses (Visionup Co., Ltd., Kyoto, Japan) were used during these exercises. These glasses have transparent lenses allowing full vision and opaque lenses that intermittently obstruct vision to create a stroboscopic effect [13,34]. In our study, a strobe frequency of 1 Hz and an opacity level of 70% were used.

**Figure 2:**
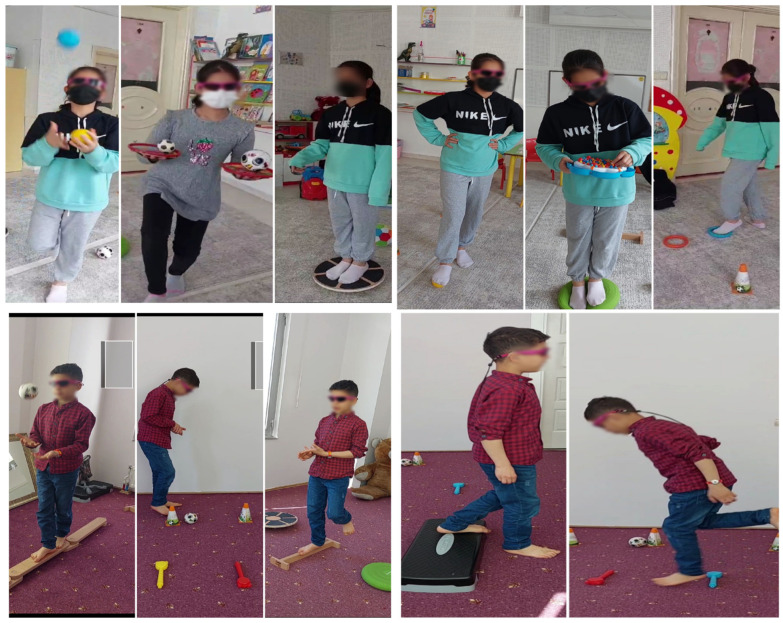
Examples of dual-task stroboscopic visual training exercises.

The dual-task stroboscopic exercise training consisted of four stages:

- Stage 1: Motor–motor dual-task exercises were performed for 10 min. One of the motor tasks involved conventional balance exercises such as standing on one leg, standing on a balance board, walking straight, walking sideways, or kicking a ball. Simultaneously, the participant was asked to maintain their position while performing a second motor task, such as holding two half-filled cups in both hands with elbows flexed at 90° and arms close to the torso, transferring an object between hands, catching a ball thrown by the therapist, or clapping.
- Stage 2: The exercises from Stage 1 were repeated for 10 min while wearing stroboscopic glasses. Before beginning the exercises with the stroboscopic glasses, participants practiced throwing and catching a ball while facing each other to familiarize themselves with the altered visual conditions.
- Stage 3: Motor-cognitive dual-task exercises were performed for 10 min. The motor tasks consisted of conventional balance exercises as described in Stage 1. Simultaneously, the participant was asked to perform cognitive tasks such as counting backward by ones from a two-digit number, reciting the months of the year in reverse order, or naming the days of the week backward.
- Stage 4: The exercises from Stage 3 were repeated for 10 min while wearing stroboscopic glasses. Before beginning the exercises with the stroboscopic glasses, participants practiced throwing and catching a ball while facing each other to familiarize themselves with the altered visual conditions [12,35] (Figure 2).

### 2.6. Statistical Analysis

All data were analyzed using SPSS version 25 (IBM Corp, Armonk, NY, USA). Results are reported as arithmetic mean ± standard deviation (X^−^ ± SD). The Kolmogorov–Smirnov test was used to assess the normality of data distribution. As the data were not normally distributed, group comparisons were performed using the non-parametric Kruskal–Wallis test. Pairwise group comparisons were performed using the Mann–Whitney U test for parameters that showed a statistically significant difference. Within-group pre- and post-treatment comparisons were conducted using the Wilcoxon Signed-Rank test. Sample size calculation was conducted using G*Power 3.1.9.7 software, indicating that a minimum of 11 participants per group was required to achieve 80% statistical power (β = 0.20) at a significance level of 0.05 (α = 0.05). Although multiple comparisons were performed in this study, no alpha correction was applied. Therefore, the results should be regarded as exploratory, and *p*-values are presented alongside effect sizes to allow a more comprehensive interpretation of the findings. To enhance clarity and maintain concise data presentation, detailed variability reporting was provided only for the GyKo parameters that demonstrated clinically meaningful differences, and these changes were described in the text.

## 3. Results

The physical and demographic characteristics of the children in the three groups were similar (*p* > 0.05) (Table 2).

When the groups were compared, significant differences were found among the groups only in the following parameters (*p* < 0.05): post-treatment PBS scores; post-treatment Distance Length (GyKoDL) during the SLS test under eyes-open conditions, and post-treatment TUG completion times. All other test results were similar among the groups before or after intervention (*p* > 0.05).

Results of the static balance assessments were comparable across all groups at both pre- and post-treatment under eyes-open and eyes-closed conditions (*p* > 0.05). However, post-treatment GyKoDL on the SLS test under eyes-open conditions was significantly higher in the DTSVT group compared to the control group (*p* < 0.05). The Tandem Romberg and SLS tests were administered for a maximum of 30 s. When both of these tests were performed under eyes-closed conditions, the average duration for which participants could maintain the position increased across all groups. However, this improvement in duration was not consistently reflected in the objective postural sway measurements recorded by the GyKo device. Specifically, the sway parameters either increased or did not decrease to a degree that reached statistical significance (*p* > 0.05) (Table 3).

When comparing pre- and post-treatment values, average durations in static balance tests generally improved across groups, except for the Tandem Romberg test in the control group, yet these within-group changes were not statistically significant (*p* > 0.05). A significant post-treatment increase was observed in the Tandem Romberg duration under eyes-closed conditions in the CBT group, and a significant increase in GyKoDL during the same test was noted in the control group (*p* < 0.05) (Table 3).

The total PBS score, which reflects both static and dynamic balance capacities, showed significantly greater post-treatment improvement in the DTSVT group compared to both the control and CBT groups (*p* < 0.05). Additionally, significant between-group differences were observed in the following parameters: pre-treatment functional reach distance, pre-treatment FSST completion time, and pre-treatment eyes-closed duration on the Balance Disc Test. Post-treatment, DL and Anterior–Posterior Mean Distance (APMD) during the FRT also differed significantly among the groups (*p* < 0.05). All other dynamic balance parameters were comparable between groups both before and after treatment (*p* > 0.05) (Table 4).

Within-group comparisons showed that FSST completion time significantly decreased from pre- to post-treatment in both intervention groups (CBT and DTSVT) (*p* < 0.05). In the DTSVT group, GyKoDL and Ellipse Area (EA) values decreased in the post-treatment FRT, while in the control group, Medial–Lateral Mean Distance (MLMD) and GyKoDL values decreased post-treatment. FRT distance significantly increased post-treatment in both the control and CBT groups (*p* < 0.05). No significant within-group differences were observed in the remaining dynamic balance parameters (*p* > 0.05) (Table 4).

FGA scores were similar across groups both before and after treatment (*p* > 0.05). Baseline 10 MWT times differed significantly among the groups (*p* < 0.05). Post-treatment, significant differences were observed among the groups in the TUG and Step Test times (*p* < 0.05). All other functional mobility parameters were comparable before and after treatment (*p* > 0.05) (Table 5).

Regarding postural sway parameters, the CBT group showed a significant reduction in DL on the Step Test post-treatment, while the control group exhibited a significant decrease in DL on the 10 MWT (*p* < 0.05). No significant within-group differences were found in other functional mobility parameters when comparing pre- and post-treatment values (*p* > 0.05) (Table 5).

Table 6 provide a consolidated overview of the outcome patterns across groups. Both intervention groups demonstrated improvements in functional balance and mobility measures (PBS, TUG, FSST), while changes in gait speed remained minimal and comparable across groups. Overall, these tables help clarify the directional trends of the results and support a more integrated interpretation of group-level changes (Table 6).

## 4. Discussion

Balance is a multifaceted concept, not solely dependent on a single neural system such as the vestibulospinal pathway, which makes its assessment and improvement inherently challenging. Our findings suggest a potential trend toward enhanced postural stability and functional mobility in children who received dual-task stroboscopic visual training compared to conventional balance training. It should be noted that multiple comparisons were conducted without alpha correction; therefore, the results should be interpreted cautiously as exploratory. Readers are encouraged to consider effect sizes alongside *p*-values. While many outcomes were not statistically significant, alterations in postural sway parameters (e.g., GyKo DL) may reflect early, preliminary intervention effects, and the non-significant findings help delineate the scope and limitations of this exploratory study.

All children in our sample had bilateral severe sensorineural hearing loss (71–90 dB) and used hearing aids. Prior reports show that children with severe hearing loss exhibit greater static balance instability than normal-hearing peers under both eyes-open and eyes-closed conditions [36]. Maes et al. similarly found that children with sensorineural hearing loss and vestibular dysfunction demonstrate the poorest balance performance [37]. Although stratifying participants by the degree of hearing loss could have offered more precise insights, our limited sample size did not allow such subgroup analyses.

We used standardized clinical balance tests—core tools for clinicians—along with objective sway parameters from the GyKo device, which quantitatively captures balance performance [38]. This combined approach may help move assessment beyond simple binary classifications toward a more nuanced grading system, although additional validation is needed. To ensure reliable testing, participants were familiarized with all tasks and provided standardized rest intervals. Although some treatment-induced changes were not evident in clinical scores, GyKo-based alterations suggest that balance may improve in ways not immediately reflected in functional measures. Our observations suggest that balance may change gradually, with initial increases in postural sway possibly reflecting early adjustments rather than deterioration. As training progresses, stability may improve and sway may decrease, which could contribute to longer durations of maintained balance, although these interpretations remain tentative. This pattern emerged across several measures: in the eyes-open SLS test, duration remained stable while the DTSVT group showed higher GyKoDL; in the eyes-closed Tandem Romberg test, the CBT group improved in duration but exhibited increased sway; and in the Functional Reach Test, sway parameters (GyKoDL, APMD) increased despite comparable reach distances. Together, these findings suggest that subtle, progressive adjustments in postural control may occur before measurable changes are detected, but this interpretation remains tentative and should be confirmed through more objective sway metrics.

Most studies in this field compare individuals who are hard-of-hearing with typically developing peers. Interventions often involve sports or standard vestibular exercises—eye-hand coordination tasks, visual-motor training, static and dynamic balance exercises, and targeted motor-skills programs [4,38,39,40,41]. A systematic review noted that most interventions shared similar characteristics, frequently involving sports such as swimming, football, volleyball, or basketball, typically delivered 2–3 times per week for 10–14 weeks. Although such programs appear promising for balance and gait, methodological limitations and low evidence quality restrict conclusions; only one study reported adverse effects [18]. Another review found that vestibular rehabilitation may improve balance and gait in children with hearing loss but emphasized the need for stronger study designs [17]. A multidimensional perspective may be helpful for understanding balance and balance-related problems, and exercise programs may need to be structured with this broader framework in mind. In our study, we adopted an innovative approach by investigating the effects of dual-task stroboscopic visual training as a balance intervention. Given that visual and perceptual abilities may be influenced by stroboscopic training, and that such training could potentially contribute to neuroplastic changes in the visual system, it may be worthwhile for training programs to consider the demands of daily life, where dual- or multi-task situations are common and single-task scenarios are comparatively rare. This supports the need for novel, methodologically robust programs beyond traditional sports-based interventions. The findings from our study are consistent with this hypothesis but do not provide definitive support. The DTSVT group demonstrated higher PBS scores and faster TUG completion times compared with the control and CBT groups. Within-group analyses showed that only the intervention groups improved significantly in FSST performance. In addition, GyKoDL values during eyes-open SLS were significantly higher in the DTSVT group than in controls. However, the lack of significant differences in other parameters likely reflects the limited sample size.

The vestibular system plays a vital role in daily life, and contributes to a broad range of functions, from reflexes to the highest levels of voluntary behavior [42]. Studies examining whether visual neuroplasticity develops in individuals who are hard-of-hearing propose that future research with adjustable stimulus difficulty and consideration of ocular dominance may yield new insights. Stroboscopic training, increasingly used in sports such as basketball and football, reduces continuous visual feedback and requires extracting information from minimal input, thereby training perceptual and attentional systems. Studies in healthy adults show benefits for anticipatory timing, visual cognition, attention, and information encoding [11,12]. While these findings highlight the potential value of visually demanding interventions, other sensorimotor modalities have also been explored. In an 8-week proprioceptive and core stability training study with a 6-month detraining period, 30 male participants who are hard-of-hearing were randomly assigned to three groups, and balance improvements persisted only in the proprioception group. [43]. By contrast, our study evaluated outcomes only immediately post-intervention, preventing conclusions about long-term retention or developmental influences.

Although we did not identify clear superiority in static vs. dynamic tasks, prior research shows vestibular dysfunction may be more easily compensated during static tasks [44]. In our results, both intervention groups improved in eyes-closed Tandem Romberg and Functional Reach, but improvements differed by group—duration-based gains in CBT and sway-based gains in DTSVT. One study found that children with congenital sensorineural hearing loss struggled most during eyes-closed SLS, underscoring vestibular involvement [45].

Hearing loss may reduce social and physical activity, leading to slower gait [46]. and is associated with poorer executive function relevant to balance [47]. Rine et al. found balance improved but gait speed remained lower in deaf children [39]. Similarly, gait speed did not improve significantly in our study, although small numerical increases occurred. This likely reflects our intervention’s emphasis on balance and visual-cognitive demands rather than gait-specific training. Studies show gait changes are more evident with proprioceptive or dual-task protocols [9]. Children with hearing loss show greater difficulty in tasks requiring heightened balance or somatosensory demand [40]. and one study reported clinically relevant differences in ground-reaction forces with slower walking speeds [48]. In our 10 MWT, slight increases in speed were not significant and may reflect cautious gait strategies.

Dual-task impairments are associated with fall risk in older adults, and dual-task training may improve motor and cognitive flexibility [15,49,50]. Deaf children show slower gait and increased lower-extremity activation under dual-task conditions, reflecting reduced mechanical efficiency [51]. In our results, DTSVT participants showed higher PBS scores, shorter TUG times, and greater GyKoDL in eyes-open SLS, with more pronounced FSST improvements. These findings suggest comparatively greater functional and postural benefits with DTSVT, though confirmation requires larger samples.

Our study has some limitations. First, the sample size was relatively small, as recruiting children who met the strict inclusion and exclusion criteria was challenging—particularly because many candidates had already undergone surgery, which limited the pool of eligible participants. Although the minimum sample size determined by GPower was achieved, larger studies are needed to enhance statistical power and generalizability. Additionally, multiple outcomes were analyzed without statistical correction (e.g., Bonferroni or FDR), which may increase the risk of Type I error. Therefore, the findings should be interpreted with caution, and future studies with larger samples should apply appropriate adjustment methods. Another limitation of this study is that the same physiotherapist conducted both the intervention and outcome assessments, which may increase performance and measurement bias. This occurred because the study centers lacked routine physiotherapy services and trained staff. We followed consistent procedures to reduce bias, and we recommend separating these roles in future research. However, to avoid bias, participants were unaware of which treatment they received, and the statistical analysis was conducted by another researcher who was unaware of the participants. Additionally, baseline differences between groups in some measures (e.g., FRT and FSST) could not be statistically adjusted, which may complicate the interpretation of post-treatment outcomes. These results should therefore be interpreted with caution, and future studies should consider stratified randomization to improve baseline equivalence. Although dual-task stroboscopic visual training may have longer-lasting effects, this cannot be determined from the present study because outcomes were assessed only immediately after treatment. This represents a limitation, as it remains unclear whether the observed improvements persist over time, which intervention produces longer-lasting effects, or to what extent the results are influenced by normal developmental progression. Future studies should include larger and more diverse samples, incorporate longer-term follow-up assessments, and use stratification based on the degree of hearing loss and vestibular function to more clearly identify which subgroups benefit most from the intervention.

## 5. Conclusions

Our findings suggest potential benefits of dual-task stroboscopic visual training in improving postural stability, gait, and functional mobility in children who are hard-of-hearing compared to conventional balance training. These results are exploratory, and effect sizes are provided to facilitate interpretation of the magnitude of observed effects. Future studies involving larger samples and children with varying hearing profiles will help improve the generalizability of these findings.

It is also important to assess balance and gait using objective methods in both clinical and laboratory settings. As demonstrated in our study, incorporating novel elements into balance training may enhance the effectiveness of exercise programs and could be adapted to other patient populations with balance impairments. We hope this study contributes to the field of hearing impairment and serves as a model for future research and training programs targeting other disability groups.

## Figures and Tables

**Table 1:** Exercises provided to the Conventional Balance Training group.

Static Exercises	Dynamic Exercises
Single-leg stance (right and left separately)	Walking forward and backward on a balance beam
Half-squat, first on both feet then on one foot	Step-ups and step-downs
Hopping on one foot	Side walking
Heel-to-toe stance on a straight line	Cross-step walking
Rolling a ball with one foot while standing on the other	Walking through obstacles
Rising onto the toes	Semi-tandem and tandem walking
Rising onto the heels	Figure-eight walking
Standing on a balance board	Walking on tiptoes and heels

**Table 2:** Comparison of the Physical Characteristics of the Groups.

	Control Group (*n* = 11)	Conventional Balance Training (CBT) Group (*n* = 9)	Dual-Task Stroboscopic Visual Training (DTSVT) Group (*n* = 8)		
	X^−^ ± SD	X^−^ ± SD	X^−^ ± SD	χ^2^	*p*
**Age (years)**	9.21 ± 2.01	8.67 ± 1.58	10.38 ± 1.41	4.323	0.115
**Height (m)**	1.33 ± 0.16	1.27 ± 0.16	1.34 ± 0.11	1.079	0.583
**Body Weight (kg)**	30.29 ± 10.45	30 ± 11.93	32 ± 6.87	1.127	0.569
**BMI (kg/m^2^)**	17.61 ± 3.26	16.85 ± 3.59	17.98 ± 1.72	0.097	0.953

Abbreviations: m, meter; kg, kilogram; BMI, Body Mass Index; X^−^ ± SD: mean ± standard deviation. Statistical significance was set at *p* < 0.05. Kruskal–Wallis H test.

**Table 3:** Within-Group and Between-Group Comparisons of Static Balance Measures.

	Within-Group			Between-Group	
Outcome Measure	Control (*n* = 11) Pre → Post Mean (*p*, Cohen’s *d*)	CBT (*n* = 9) Pre → Post Mean (*p*, Cohen’s *d*)	DTSVT (*n* = 8) Pre → Post Mean (*p*, Cohen’s *d*)	*p* (Pre, Post)	Summary
Romberg EO (sec)	29.29 → 28.73 (*p* = 0.66, *d* = 0.18)	29.11 → 30.00 (*p* = 0.32, *d* = 0.33)	26.25 → 30.00 (*p* = 0.16, *d* = 0.54)	Pre: 0.50 Post: 0.46	All groups similar; DTSVT shows numerically largest gain
GyKoEA (Romberg EO)	634 → 400 (*p* = 0.25, *d* = 0.38)	264 → 733 (*p* = 0.95, *d* = 0.31)	637 → 473 (*p* = 0.67, *d* = 0.15)	Pre: 0.92 Post: 0.96	No meaningful change
Romberg EC (sec)	16.43 → 18.64 (*p* = 0.55, *d* = 0.13)	16.78 → 20.67 (*p* = 0.04 *, *d* = 0.75)	15.88 → 21.88 (*p* = 0.08, *d* = 0.66)	Pre: 0.93 Post: 0.74	CBT and DTSVT show improvements; CBT significant
GyKoEA (Romberg EC)	1307 → 1331 (*p* = 0.93, *d* = 0.11)	703 → 1331 (*p* = 0.14, *d* = 0.48)	1108 → 648 (*p* = 0.78, *d* = 0.23)	Pre: 0.83 Post: 0.18	No consistent pattern
Single-Leg Stance EO (sec)	18 → 20.82 (*p* = 0.29, *d* = 0.32)	23.67 → 24.56 (*p* = 0.59, *d* = 0.14)	23.88 → 27.25 (*p* = 0.11, *d* = 0.69)	Pre: 0.43 Post: 0.42	DTSVT shows largest improvement trend
GyKoEA (SLS EO)	875 → 767 (*p* = 0.48, *d* = 0.12)	389 → 1100 (*p* = 0.07, *d* = 0.54)	825 → 736 (*p* = 0.89, *d* = 0.30)	Pre: 0.42 Post: 0.77	No meaningful difference
Single-Leg Stance EC (sec)	6.29 → 8.82 (*p* = 0.59, *d* = 0.27)	6.56 → 8.56 (*p* = 0.26, *d* = 0.42)	8 → 12 (*p* = 0.09, *d* = 0.69)	Pre: 0.40 Post: 0.37	DTSVT shows largest gain trend under EC condition
GyKoEA (SLS EC)	1592 → 1196 (*p* = 0.59, *d* = 0.24)	1392 → 1753 (*p* = 0.21, *d* = 0.48)	1858 → 1380 (*p* = 0.78, *d* = 0.40)	Pre: 0.90 Post: 0.47	No meaningful change

* Statistically significant at *p* < 0.05. EA: Total Sway Area, cm^2^, EC: Eyes Closed, EO: Eyes Open.

**Table 4:** Within-Group and Between-Group Comparisons of Pediatric Balance Scale Scores and Dynamic Balance Measures.

	Within-Group			Between-Group	
Outcome Measure	Control (*n* = 11) Pre → Post Mean (*p*, Cohen’s *d*)	CBT (*n* = 9) Pre → Post Mean (*p*, Cohen’s *d*)	DTSVT (*n* = 8) Pre → Post Mean (*p*, Cohen’s *d*)	*p* (Pre, Post)	Summary
Pediatric Balance Scale (PBS)	54.64 → 54.70 (*p* = 1.00, *d* = 0.05)	54.44 → 55.00 (*p* = 0.16, *d* = 0.47)	54.75 → 55.88 (*p* = 0.10, *d* = 0.89)	Pre: 0.77 Post: 0.03 *	DTSVT > Control = CBT
Functional Reach Test (FRT)	23.79 → 27.36 (*p* = 0.03 *, *d* = 0.75)	22 → 26.11 (*p* = 0.01 *, *d* = 1.82)	26.13 → 29.75 (*p* = 0.11, *d* = 0.70)	Pre: 0.01 * Post: 0.06	Control = CBT > DTSVT
GyKoEA (FRT)	3614 → 2357 (*p* = 0.25, *d* = 0.26)	2682 → 2004 (*p* = 0.67, *d* = 0.04)	3629 → 1838 (*p* = 0.04 *, *d* = 0.97)	Pre: 0.34 Post: 0.09	DTSVT shows largest sway reduction
Four Square Step Test (FSST)	10.29 → 9.45 (*p* = 0.23, *d* = 0.35)	10.67 → 8.78 (*p* = 0.04 *, *d* = 0.93)	9.25 → 7.88 (*p* = 0.03 *, *d* = 1.06)	Pre: 0.02 * Post: 0.15	DTSVT > Control
GyKoEA (FSST)	1884 → 1853 (*p* = 0.66, *d* = 0.15)	2256 → 2103 (*p* = 0.59, *d* = 0.16)	1977 → 2003 (*p* = 1.00, *d* = 0.05)	Pre: 0.59 Post: 0.91	No meaningful difference
Balance Disc Test (EO)	22.5 → 25.91 (*p* = 0.23, *d* = 0.37)	17.67 → 25.0 (*p* = 0.09, *d* = 0.65)	25.63 → 28.75 (*p* = 0.36, *d* = 0.37)	Pre: 0.08 Post: 0.67	Mild gains in CBT and DTSVT
GyKoEA (BD EO)	2251 → 1747 (*p* = 0.48, *d* = 0.34)	1608 → 1217 (*p* = 0.44, *d* = 0.32)	1183 → 1259 (*p* = 0.67, *d* = 0.05)	Pre: 0.50 Post: 0.64	No meaningful change
Balance Disc Test (EC)	7.79 → 9.36 (*p* = 1.00, *d* = 0.11)	7.89 → 7.44 (*p* = 0.86, *d* = 0.16)	10.75 → 15.25 (*p* = 0.25, *d* = 0.45)	Pre: 0.04 * Post: 0.18	DTSVT > Control at baseline
GyKoEA (BD EC)	2719 → 2364 (*p* = 0.29, *d* = 0.10)	2575 → 2941 (*p* = 0.95, *d* = 0.11)	1649 → 1531 (*p* = 0.78, *d* = 0.12)	Pre: 0.41 Post: 0.64	No meaningful change

* Statistically significant at *p* < 0.05. EA: Total Sway Area, cm^2^, EC: Eyes Closed, EO: Eyes Open.

**Table 5:** Within-Group and Between-Group Comparisons of Gait and Functional Mobility Assessments.

	Within-Group			Between-Group	
Outcome Measure	Control (*n* = 11) Pre → Post Mean (*p*, Cohen’s *d*)	CBT (*n* = 9) Pre → Post Mean (*p*, Cohen’s *d*)	DTSVT (*n* = 8) Pre → Post Mean (*p*, Cohen’s *d*)	*p* (Pre, Post)	Summary
Timed Up and Go Test (sec)	10 → 9.09 (*p* = 0.06, *d* = 0.66)	10.33 → 10.25 (*p* = 0.89, *d* = 0.00)	9.38 → 8.25 (*p* = 0.07, *d* = 0.73)	Pre: 0.22 Post: 0.01 *	DTSVT > Control = CBT
GyKoEA (TUG)	2588 → 2608 (*p* = 0.86, *d* = 0.05)	1792 → 1895 (*p* = 0.68, *d* = 0.08)	2192 → 3565 (*p* = 0.16, *d* = 0.69)	Pre: 0.33 Post: 0.24	No meaningful difference
10-Meter Walk Test (sec)	6.21 → 5.64 (*p* = 1.00, *d* = 0.18)	7.11 → 6.22 (*p* = 0.16, *d* = 0.53)	6.25 → 5.75 (*p* = 0.52, *d* = 0.28)	Pre: 0.03 * Post: 0.23	No clear advantage
GyKoEA (10 MWT)	851 → 1128 (*p* = 0.05, *d* = 0.60)	623 → 633 (*p* = 0.68, *d* = 0.03)	927 → 933 (*p* = 0.58, *d* = 0.02)	Pre: 0.41 Post: 0.53	No meaningful change
Step Test (sec)	3.21 → 3.18 (*p* = 0.56, *d* = 0.17)	3.22 → 2.89 (*p* = 0.18, *d* = 0.47)	3.75 → 2.75 (*p* = 0.06, *d* = 0.59)	Pre: 0.13 Post: 0.01 *	DTSVT > others (post-treatment)
GyKoEA (Step)	1752 → 1781 (*p* = 1.00, *d* = 0.01)	1735 → 1362 (*p* = 0.14, *d* = 0.46)	1342 → 1681 (*p* = 0.33, *d* = 0.45)	Pre: 0.10 Post: 0.79	No meaningful difference

* Statistically significant at *p* < 0.05. EA: Total Sway Area, cm^2^.

**Table 6:** Directional Summary of Group-Level Changes in Balance and Gait Outcomes (Non-Sway Outcomes).

Outcome	Control	CBT	DTSVT	Interpretation
Pediatric Balance Scale (PBS)	→	↑	↑	Balance improvement in both intervention groups; DTSVT highest post-treatment score
Romberg EO (sec)	→	→	↑	Minor improvement appears only in DTSVT; groups largely similar
Romberg EC (sec)	→	↑	↑	Eyes-closed stability improved in both intervention groups
Single-Leg Stance EO (sec)	→	→	↑	DTSVT shows better ability to maintain stance with visual input
Single-Leg Stance EC (sec)	→	→	↑	Improvements mainly in DTSVT under high sensory challenge
Balance Disc Test (EO)	→	↑	↑	Slight improvement in intervention groups; not statistically strong
Balance Disc Test (EC)	→	→	↑	DTSVT showed most notable improvement under vestibular load
Timed Up and Go Test (sec)	→	↑	↑	Increased functional mobility in intervention groups
Four Square Step Test (sec)	→	↑	↑	Dynamic balance benefits, more pronounced in DTSVT
10-Meter Walk Test (10 MWT)	→	→	→	No meaningful change in gait speed
Functional Reach Test (FRT)—Distance	↑	↑	→	Distance improved mainly in Control and CBT; DTSVT stable
Step Test (sec)	→	↑	↑	Both interventions improved step performance; DTSVT showed the largest improvement

## Data Availability

The data presented in this study are available on request from the corresponding author due to privacy and ethical restrictions related to research involving children who are hard-of-hearing.
